# Annotations

**Published:** 1911-08-05

**Authors:** 


### Annotations.

### The Cause of Cancer.

The report issued by the Cancer Research Fund again comments upon two points comforting to the general public, that cancer is not catching and it may occasionally be recovered from. Apparently a considerable number of cases of spontaneous healing of malignant new growths have been observed in mice. Many cases in which there was no doubt as to the diagnosis of the disease have been recorded in the human being in which the patient has recovered perfect health. We have known of a case in which after removal of the breast for cancer a secondary growth occurred in one eye, for which the eye was removed. The liver then enlarged and nodules of secondary growth could be felt upon its surface. Yet the patient, although greatly wasted at this time, eventually recovered. The liver returned to its former size and the normal weight was regained.

### The Milk Problem.

In cities people must depend on outside supplies for their milk. On general principles one expects better milk from cows that live in the open pastures of the country than from those whose lives are passed—as until lately was often the case—in dark and ill-ventilated stables in the poorer parts of a town. Against this must be set, however, certain other factors. Inspection, both of the cows and the conditions of milking, may be more careless in the country than it is in the town. Also, if the milk is to be conveyed for a considerable distance without getting sour, some preservative must be put into it. It is conceded that a little of this is necessary, but many farmers put in so much that the preservative in its turn becomes a means of doing harm. Observers have noted that microbes are fewer in number in the milk sold in the poorer parts of towns than in the richer, and have surmised that this is due not to the greater purity of the milk but to the greater amount of boric acid, or some similar substance which has been put in to keep it from souring. Consequently they have inferred that much of the infantile trouble which is diagnosed as summer diarrhœa is really irritant poisoning due to the preservatives in the milk given to children. These difficulties, which are of annual, if not of absolutely chronic occurrence, are intensified in a summer like this, when the long drought has lessened the supply of milk. To make up the deficiency foreign milk has been imported, some preserved, but some in its natural condition. Concerning this the means of inspection are even more uncertain than with that of native production. So a few years ago an outbreak in a watering-place in the island of Arran was traced to infection from canned milk which had been brought in to supplement the deficiencies of the native supply, a fact which shows that even the heat required for the condensation of milk is not always strong enough to destroy all germs. These things give cause for thought—not of a wholly comforting sort. But one thing we may remember—that of all the things that are needed to sustain life, milk is the one which most needs to be produced as near as possible to the place where it is consumed. We can import grain safely, and the safeguarding of foreign meat and fruit is a by no means impossible task. But milk stands in another category. We may desire our people to go back to the land, and dream of a self-supporting England, but the fact remains that we have a great urban population which must be fed. We cannot in this island provide all their food, but one thing it is necessary that we should provide at home—milk, the essential food of childhood, the desirable food of every age—milk produced under sound supervision which will carry it safely from cow to consumer.

### A New Professional Organisation.

Amid the stress and turmoil of the discussion on the Insurance Bill, a new organisation seeks the suffrages and the subscriptions of the medical profession. It has appropriated the ambitious title of the "Imperial Medical Reform Union," and has adopted as its official mouthpiece a weekly paper already in existence, the *Medical Times*. The Reform Union announces that it does not compete with the British Medical Association, but that there is room for both. In electing Dr. Cursham Corner to the presidential chair, the Union has begun well, for he is well acquainted with the conditions of general practice in those districts of large towns which will be most affected by the Bill. Time will show whether the officers of the Union are to be the pioneers of a new and brighter era in medical practice, or whether they have underrated the difficulties of their task and overrated their capacity for overcoming them. The newly-born Union will need to be an infant Hercules indeed if it is to grapple successfully with the problems at present agitating the profession.

### The Prohibition of Absinthe in France.

Attached to the report presented to the French Senate by the Commission appointed to examine the new law to prohibit the manufacture and sale of absinthe in France, are statistics showing the consumption of this drink. This consumption has grown from 6,713 hectolitres in 1873 to 18,000 hectolitres in 1880, to 105,258 hectolitres in 1890, and to 238,467 hectolitres in 1900. Since this last date it has gradually decreased until in 1910 it was only 172,166 hectolitres. It is probable that much time will elapse before the new law will pass the Chamber of Deputies, partly because of the difficulty of finding revenue to replace that lost by the suppression of absinthe. Meanwhile, however, the great increase in absinthe drinking since 1873 is disquieting, for there can be little doubt that the increase is to be found chiefly among the working-classes. It is unfortunate that increased prosperity among the French working-classes should tally with marked increases in the consumption of the "little green devil."

